# Improved tumour marker sensitivity in detecting colorectal liver metastases by combined type IV collagen and CEA measurement

**DOI:** 10.1007/s13277-015-3729-z

**Published:** 2015-07-11

**Authors:** Hanna Nyström, Björn Tavelin, Moa Björklund, Peter Naredi, Malin Sund

**Affiliations:** 1 Department of Surgical and Perioperative Sciences, Surgery, Umeå University, 90185 Umeå, Sweden; 2 Department of Radiation Sciences, Oncology, Umeå University, Umeå, Sweden; 3 Department of Surgery, Institute of Clinical Sciences, Sahlgrenska Academy, University of Gothenburg, Gothenburg, Sweden

**Keywords:** Colorectal liver metastases, Circulating biomarkers, CEA, Type IV collagen, Extracellular matrix

## Abstract

Carcinoembryonic antigen (CEA) is the best circulating tumour marker for colorectal liver metastasis (CLM) but has suboptimal sensitivity and specificity. Circulating type IV collagen (COLIV) is a new potential CLM marker. Here, COLIV and CEA were measured in patients with resectable CLM. COLIV levels were also related to the type of CLM. The prognostic value of these markers and the type of CLM on survival was evaluated. Preoperative plasma samples (*n* = 94) from patients (*n* = 85) with CLM undergoing liver resection were used. Seven patients underwent repeated liver resection. Samples from 118 healthy individuals served as control. Samples after liver resection (*n* = 27) were analysed and related to recurrence. COLIV and CEA levels were analysed, and the type of CLM was classified using paraffinated tissue. Results were analysed by logistic regression and receiver operating characteristic (ROC) curve analysis. CLM patients had significantly elevated levels of COLIV compared to controls (*p* = 0.001). The sensitivity of COLIV was not better than CEA, but improved sensitivity for detecting CLM was observed with a combination of the two markers compared to using either marker alone (*p* = 0.001). Circulating COLIV was elevated in 81 % and CEA in 56 % of CLM patients at diagnosis, and high marker levels were related to poor survival. In follow-up samples (*n* = 27), patients with CLM recurrence (*n* = 14) had significantly elevated COLIV levels compared to patients without postoperative recurrence (*n* = 10) (*p* = 0.001). COLIV is a promising tumour marker for CLM and can possibly be used to detect postoperative CLM recurrence. The combination of COLIV and CEA is superior to either marker alone in detecting CLM.

**Electronic supplementary material:**

The online version of this article (doi:10.1007/s13277-015-3729-z) contains supplementary material, which is available to authorized users.

## Introduction

Circulating tumour markers can be used as tools for detecting disease, defining prognosis and predicting the effect of treatment and in the follow-up and surveillance of cancer patients. For colorectal cancer (CRC) and colorectal liver metastases (CLM), the most commonly used circulating biomarker is carcinoembryonic antigen (CEA). This biomarker is mainly used to detect recurrence of CRC and not for screening due to low sensitivity and specificity [1, 2]. At CRC recurrence, 50–60 % of patients have increased levels of CEA and this tumour marker is more sensitive in detecting CLM and lymph node metastases than a local recurrence or lung metastases [1–3]. CEA levels are also frequently elevated in other forms of cancers as well as several benign conditions such as inflammatory bowel disease, pancreatitis and benign liver disease and among smokers [4, 5]. Other circulating tumour markers have been presented, but none has yet fulfilled the criteria of high sensitivity and specificity, combined with a stable and reproducible method of analysis and measurements from an easily accessible compartment, such as blood or urine.

Type IV collagen is the most abundant basement membrane (BM) protein in the human body. Previous studies have shown that type IV collagen in plasma is elevated in patients with inoperable CLM and that the levels reflect the tumour burden in the liver [6]. On the contrary, patients with primary CRC (TNM stage I–III) have normal circulating levels of this protein, which indicates that the observed increase in circulating type IV collagen is related to the CLM process. This is further underlined by the finding of highly increased type IV collagen expression in the stroma of CLM tissue [6]. In addition, type IV collagen expression in the vicinity of the cancer cells in primary CRC was also found to be a risk factor for development of a subsequent CLM [7].

Different types of CLM can be classified based on the growth pattern of the metastases in the liver, namely the *desmoplastic*, *pushing* and *replacement* types of CLM [8]. The desmoplastic type of CLM has a fibrotic capsule rich in type IV collagen and inflammatory cells, whereas the pushing type of CLM is devoid of a desmoplastic reaction and inflammatory infiltrates and only has low expression of type IV collagen in the tumour-liver interface. The pushing type of CLM is associated with a poor long-term survival, in contrast to the desmoplastic type of CLM [7–11]. The replacement type of CLM is characterized by tumour cells growing within preserved sinusoids of the liver, with minor inflammation, angiogenesis and no desmoplastic reaction.

The first aim of this study was to determine the circulating type IV collagen levels in patients with surgically treated CLM and to analyse whether this marker can be used as a method to detect disease relapse. Secondly, we wanted to study whether circulating levels of type IV collagen are related to other clinical and prognostic parameters and to the type of CLM, as there are significant differences in the expression of this protein between the different CLM growth patterns [7]. The third aim was to investigate if type IV collagen is superior to CEA in detecting CLM. The fourth aim was to analyse whether a combination of both circulating markers would increase the sensitivity for detecting CLM. The final aim was to investigate if the levels of these two markers correlated to survival.

## Methods

In the present study, the analysis was performed with the ambition to adhere to the REMARK guidelines. The project in relation to these guidelines is shown in Supplementary Table 1.

### Patient cohort and sample collection

**Fig. 1:**
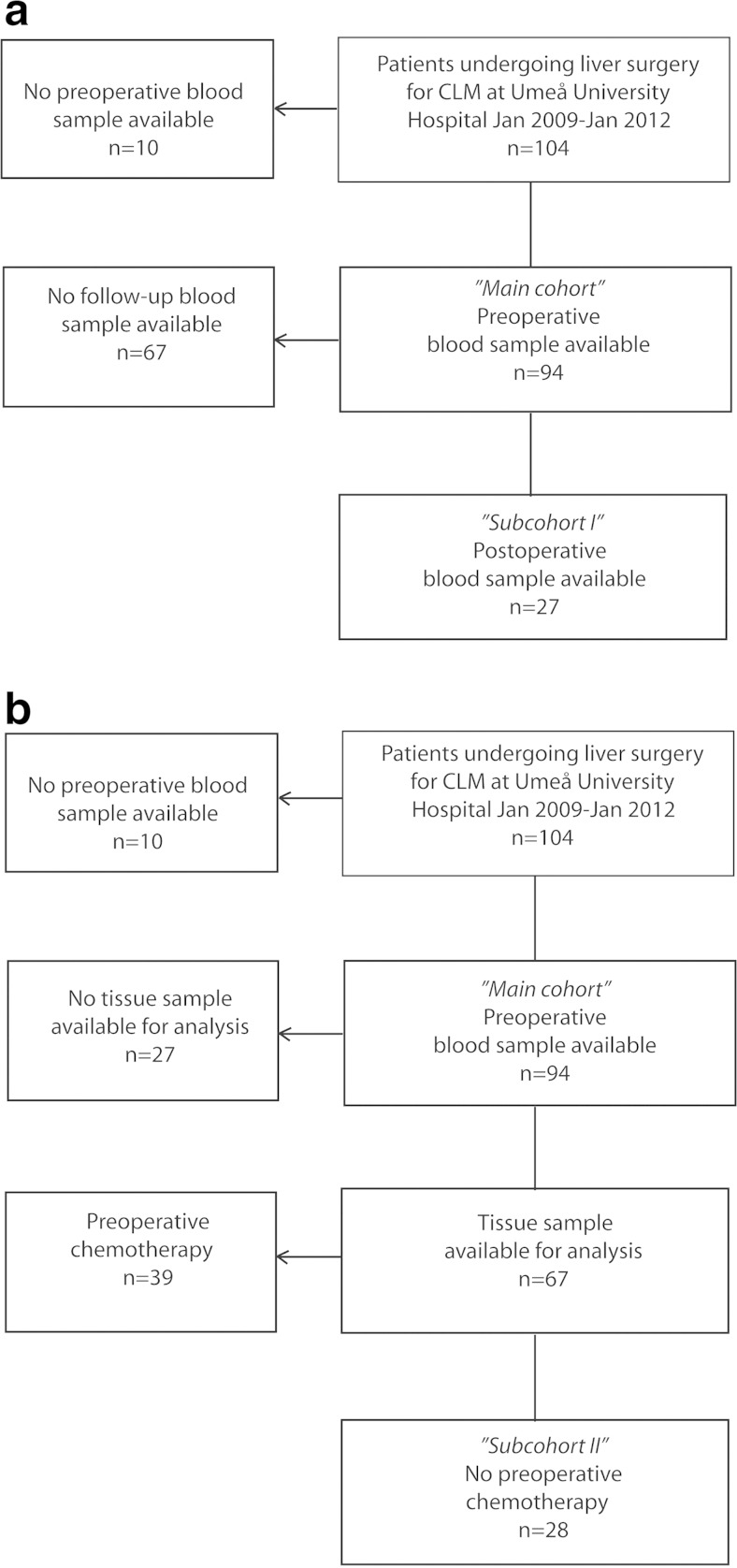
**a** Flowchart of patients included and excluded in the analysis of circulating type IV collagen in patients with colorectal liver metastases (CLM). **b** Flowchart of patients included and excluded in the analysis of growth pattern of CLM

Ninety-four preoperative plasma samples from a cohort of 85 consecutive individual patients who underwent surgery for CLM at Umeå University Hospital between January 2009 and January 2012 were used (*main cohort*) (Fig. 1a). Seven patients in the cohort underwent repeated liver resections due to CLM relapse (three patients had two liver resections preformed, and one patient had three liver resections). The plasma sample was collected within 2 weeks prior to liver surgery. The postoperative follow-up was done according to standard protocols with clinical and radiological follow-up every 3–6 months for 2 years and then every 6–12 months up to 5 years.

For 27 (29 %) patients, a postoperative plasma sample was collected during March 2012 (referred to as *subcohort I*). These postoperative samples were collected between 6 and 42 months after liver surgery. At that time, it was not known whether these patients had a recurrence or not; this information was collected separately after the sample was retrieved.

As controls, plasma samples from a group of 118 healthy, non-smoking individuals were used. These individuals donated blood samples during population-based voluntary health controls between 1990 and 2007, and the samples were stored in the prospective biobank of Västerbotten County Council and Umeå University. All plasma samples were frozen and stored at −80 °C until analysis. Clinical data were collected from patient charts (age, gender, TNM stage of primary CRC, preoperative CEA levels at the time of CLM surgery, oncological treatment, time interval between CRC and CLM, size of the largest CLM and number of CLM).

### Assays for measurement of circulating type IV collagen and CEA

Circulating levels of type IV collagen in serum were measured using the serum Collagen IV ELISA kit (Argutus Medical, Dublin, Ireland). The samples were analysed in duplicates according to the manufacturers’ protocol. This kit is based on a sandwich ELISA using two monoclonal antibodies (clones 4H12 and ID3) directed against the 7S and collagenous domain of type IV collagen [12].

Circulating CEA levels in the control group were measured by a Luminex-based method using a commercially available human circulating cancer biomarker magnetic bead panel (Merck Millipore, Billerica, MA, USA). The samples were analysed in duplicates according to the manufacturers’ protocol.

### Tissue staining and classification of CLM type

Paraffinated sections of the largest CLM from each patient were selected, with the largest interface between tumour-liver parenchyma present. Both haematoxylin and eosin stain and a chemical reticular stain were used according to earlier descriptions [8–13, 7].

Two researchers (HN and MB) independently evaluated the samples. The types of metastasis were classified into *desmoplastic*, *pushing* or *replacement* types of CLM according to previous descriptions [6, 7]. The desmoplastic type of CLM is characterized by the presence of a thick desmoplastic rim, rich in inflammatory cells, separating the CLM from the normal liver. The pushing type of CLM grows with the tumour cells pushing the liver parenchyma away with only little desmoplasia and inflammation present at the tumour border. The replacement type of CLM is characterized by tumour cells growing within the normal architecture of the liver, with preserved sinusoids and no desmoplastic reaction or substantial inflammation present. If multiple growth patterns were present, the CLM type was classified as *mixed*. The growth patterns were classified in both the main cohort (*n* = 94) and also analysed separately in a group of patients (*n* = 28) (referred to as *subcohort II*, Table 1, Fig. 1b). The patients in subcohort II did not receive any kind of chemotherapy for at least 3 months prior liver resection and could thus be used to indicate whether chemotherapy influenced the CLM growth patterns.

**Table 1:** Patient characteristics

	Main cohort	Subcohort I	Subcohort II	Controls (*n* = 118)
	Preoperative sample (*n* = 94)	Postoperative sample (*n* = 27)	No chemotherapy (*n* = 28)	
Gender (M/F)	63/31	18/9	18/10	58/60
Age (mean ± SD)	65.3 ± 8.6	66.2 ± 7.5	69.9 ± 8.3	54.6 ± 10.3
CRC location (colon/rectum)	50/44	15/12	15/13	–
T stage CRC				
1	3	1	3	–
2	7	1	4	
3	44	12	15	
4	20	5	2	
Unknown	20	8	4	
N stage CRC				
N0	23	8	10	–
N1	23	5	10	
N2	33	8	7	
Unknown	15	6	1	
Type IV collagen (ng/ml; mean ± SD)	170.1 ± 72.5	–	155.4 ± 80.6	104.4 ± 33.0
Recurrent CLM	–	214.0 ± 145.4	–	–
Disease-free	–	82.4 ± 13.7	–	–
CEA (ng/ml; median, range)	7.6 (0.8–2073)	7.7 (1.2–200)	11.7 (0.8–96)	1.3 (0.2–4.2)
Time interval CRC-CLM in months (median, range)	0 (0–30)	0 (0–30)	10 (0–30)	–
Synchronous/metachronous CLM^a^	62/32	20/7	9/19	–
Type of CLM				
Pushing	36	8	15	–
Desmoplastic	20	12	8	
Replacement	2	0	1	
Mixed	9	2	4	
Not classifiable	16	2	0	
Unknown	11	3	–	

^a^Synchronous CLM here defined as CLM diagnosed within 6 months from diagnosis of primary tumour

### Statistical analysis

SPSS version 19 and STATA version 11 software were used for statistical analysis. Mann-Whitney *U* test was used to estimate differences between the groups, since data was not normally distributed. The inter-rater agreement regarding the classification of CLM growth pattern was calculated by using Cohen’s kappa coefficient. The probability of CLM was calculated using a logistic regression model, and the estimated probabilities were used in a receiver operating characteristic (ROC) analysis to calculate the area under the curve (AUC) for different models. In the ROC analysis for the tumour markers, every operation was included as an event, and subsequently, the samples for the seven patients that underwent repeated liver resection were here treated as independent observations (*n* = 94).

When estimating the prognostic value of the tumour markers in relation to survival, or the metastatic pattern of CLM in relation to survival, the start for the observation was set to the time of the first surgical intervention, whether patients underwent one or more than one liver resection. Thus, for the seven patients that underwent repeated liver resections, only the first preoperative sample collected at the first resection was used and the patients with missing CEA values (*n* = 6) were excluded (total events included *n* = 79). When calculating any relation between mortality and levels of circulating type IV collagen and/or CEA, patients were grouped as follows: type IV collagen and CEA both below median, one tumour marker above the median or both markers above the median. Differences in survival probability between groups were tested using the log-rank test. The level of significance was set to 5 % for all tests.

## Results

### Characterization of the cohort

Patient characteristics and oncological treatment are presented in Table 1 and the flowchart of the cohort with included and excluded patients in Fig. 1.

There was no gender difference between the main cohort and subcohort I and II (*p* = 0.964). There was a difference in gender distribution between controls and the main cohort (*p* = 0.02), and the controls were younger than the CLM patients (*p* = 0.01).

The majority of the patients received oncological treatment for their CLM prior liver surgery (neoadjuvant or conversion therapy) (*n* = 53, 56 %, Table 2). Standard regimens with intravenous FLOX (5-fluorouracil, leucovorin and oxaliplatin), FLV (5-fluorouracil, leucovorin) and FLIRI (5-fluorouracil, irinotecan) were used and in some cases also combined with antibody-based targeted treatment (bevacizumab, cetuximab and panitumumab). The majority of patients with rectal cancer were given radiotherapy (*n* = 27, 61 %). Rectal cancer with locally advanced primary tumours were usually treated with neoadjuvant oral capecitabine or intravenous FLOX in combination with radiotherapy (*n* = 19, 20 %). Fifteen patients underwent liver surgery before the removal of the primary tumour (“liver first”). The median follow-up time was 26.4 months for the study cohort.

**Table 2:** Oncological treatment

	CLM (*n* = 94)	Subcohort I (*n* = 27)
Radiotherapy	27 of 44	8 of 12
Chemotherapy CRC		
None	26	7
Neoadjuvant	15	8
Adjuvant	38	10
Conversion therapy	12	1
Unknown	3	1
Chemotherapy CLM		
None	30	6
Neoadjuvant	41	17
Adjuvant	8	2
Conversion therapy	12	1
Unknown	3	1
Biological treatment		
Bevacizumab	3	1
Cetuximab	5	3

### High preoperative circulating levels of type IV collagen in patients with CLM

**Fig. 2:**
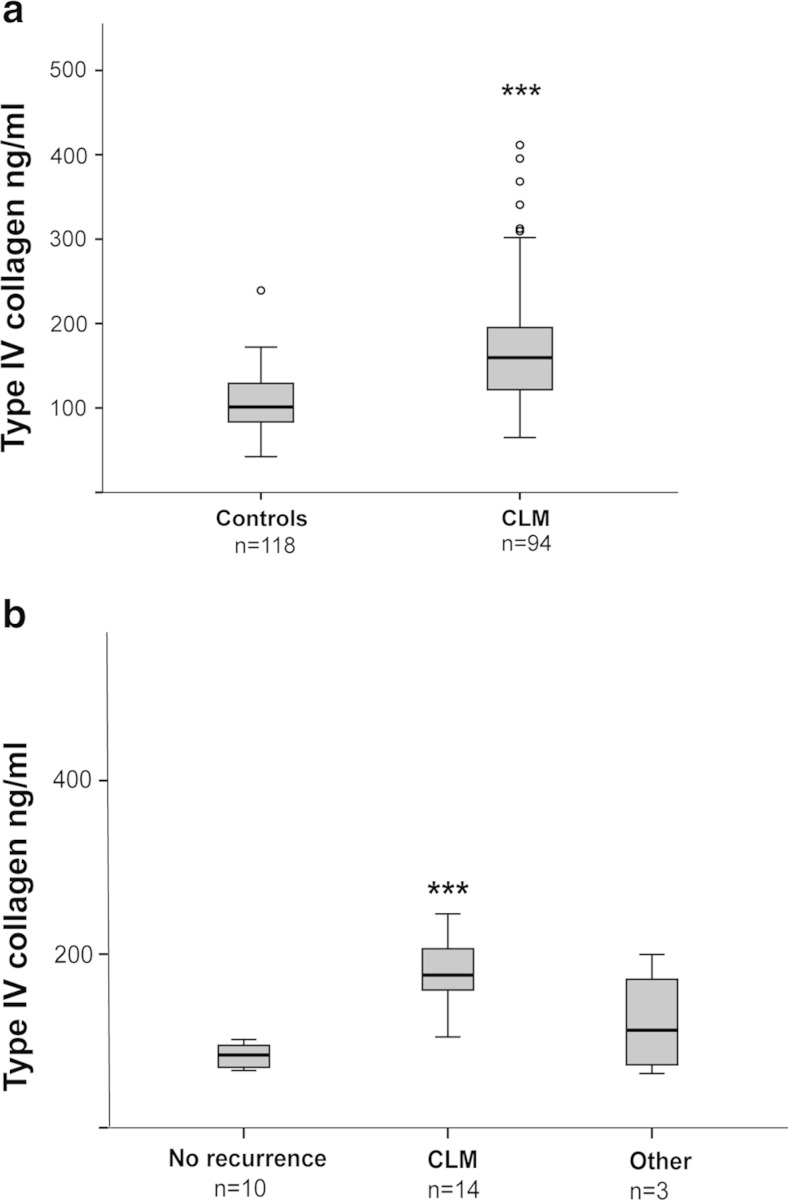
**a** Circulating type IV collagen in healthy controls compared to preoperative samples from patients with CLM. Patients with CLM had significantly higher levels of circulating type IV collagen (170.2 ± 72.5 ng/ml) compared to controls (104.3 ± 33 ng/ml) (***p = 0.001). **b** Circulating levels of type IV collagen in patients with a postoperative follow-up sample 6–42 months after liver surgery for CLM. Patients with no recurrence (*n* = 10), recurrence of CLM (*n* = 14) or with a non-liver recurrence (*n* = 3). Patients with recurrent CLM had significantly higher type IV collagen levels (214 ± 145 ng/ml) compared to the patients with no recurrence (82.3 ± 13.7 ng/ml) (***p = 0.001). The *circles* represent the outliers/ samples that are outliers outside of the whiskers

Patients with CLM (*n* = 94) had significantly higher preoperative levels of circulating type IV collagen (170.2 ± 72.5 ng/ml) when compared to healthy controls (*n* = 118) (104.3 ± 33 ng/ml) (*p* = 0.001) (Fig. 2a). There were no significant correlations between the circulating levels of type IV collagen in the CLM group and gender (*p* = 0.89), age (*p* = 0.32), the localization of the primary CRC (*p* = 0.48), T stage (*p* = 0.69), N stage (*p* = 0.13), CEA levels (*p* = 0.14), size of the largest CLM (*p* = 0.14), number of CLM (*p* = 0.075) and time interval from CRC to CLM (*p* = 0.09). There was no relation between CEA and age (*p* = 0.10) or gender (*p* = 0.35) in the CLM group.

In the control group, there was a small but significant difference in the circulating type IV collagen levels related to age (*p* = 0.001), but not correlation between CEA and age (*p* = 0.46). Neither CEA nor type IV collagen levels were related to gender in the control group (*p* = 0.76 and *p* = 0.98, respectively). Levels of the two tumour markers in relation to age and gender for both groups are shown in Supplementary Table 2a, b.

### High levels of collagen IV in patients with recurrent CLM

The 27 patients with a long-term follow-up postoperative sample (subcohort I) were divided into a group with recurrent disease (*n* = 17) and patients with no evidence of recurrent disease (*n* = 10). Patient characteristics are found in Table 1. All measured values of type IV collagen, recurrent disease and the site of recurrence for subcohort II are shown in Supplementary Table 3. Patients with recurrent disease (*n* = 17), including all types of recurrences, had significantly higher levels of type IV collagen (200.1 ± 136.9 ng/ml), compared to the patients with no recurrent disease (*n* = 10) (82.3 ± 13.7 ng/ml) (*p* = 0.001) (Fig. 2b).

Fourteen (83 %) of the patients with recurrent disease had CLM and presented with high collagen levels (214 ± 145 ng/ml) compared to the group with no recurrent disease (82.3 ± 13.7 ng/ml) (*p* = 0.001). The three patients (18 %) with a non-liver recurrence had a local rectal recurrence with a lymph node metastasis, unclassified ovarian mass or lung metastasis only (Fig. 2b).

### Preoperative circulating type IV collagen versus CEA levels in patients with CLM

**Fig. 3:**
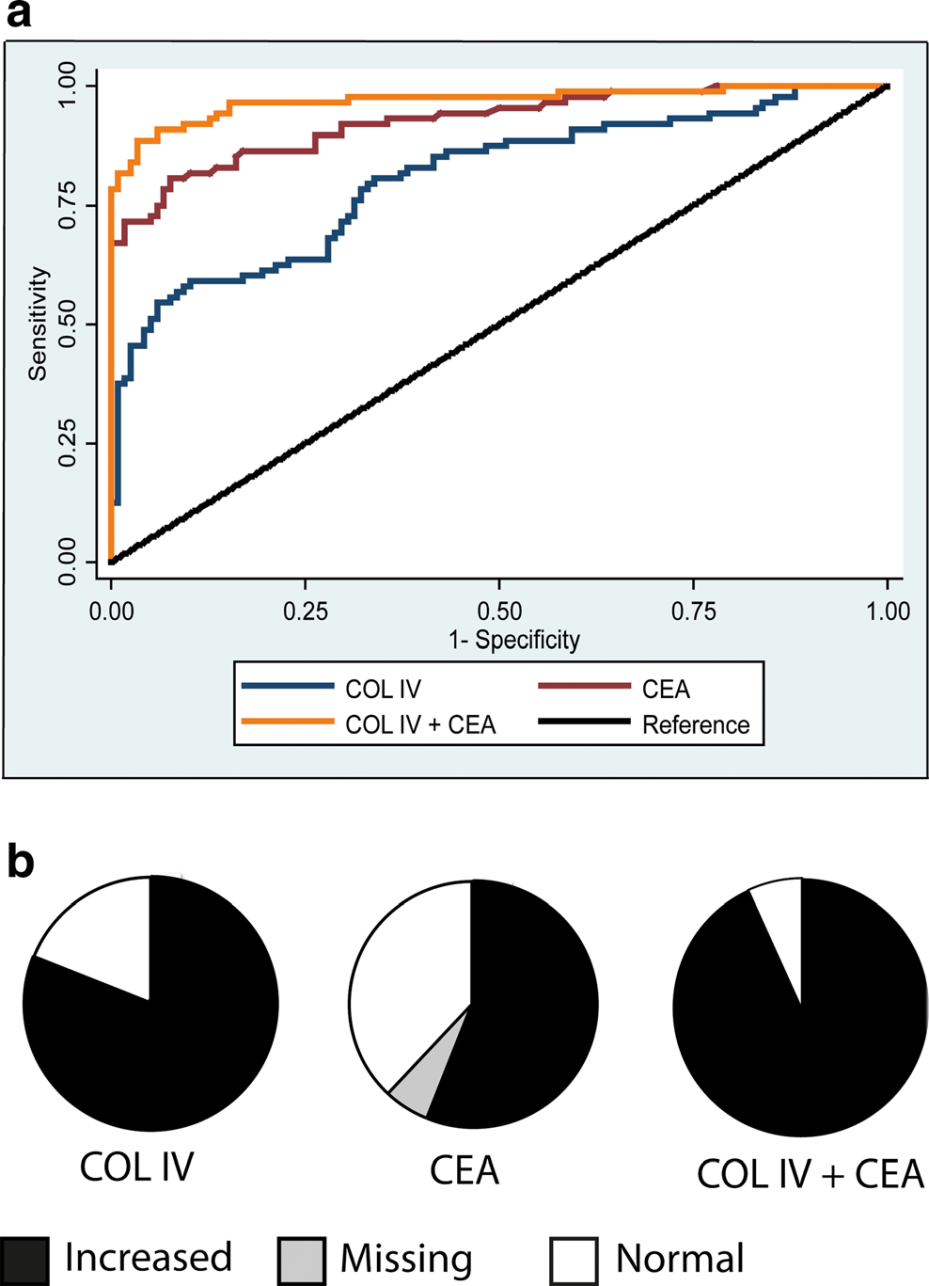
**a** ROC curve analysis of type IV collagen, CEA and a combination of both markers. Based on logistic regression, the area under the curve (AUC) was compared and the combination of markers is significantly better then CEA or type IV collagen alone (*p* = 0.001 and *p* < 0.001, respectively). **b** Comparison of percentage of CLM patients with elevated preoperative levels of type IV collagen (COL IV) and CEA and percentage of patients with at least one marker elevated. Type IV collagen levels were increased above cutoff (115 ng/ml) in 81 % of patients with CLM compared to only 56 % of patients presenting with elevated levels of CEA (cutoff 5 ng/ml). Ninety-three percent of the patients presented with at least one of the markers above these levels

The ROC analysis revealed that type IV collagen is not superior to CEA alone in detecting CLM (Fig. 3a). When combining both markers as independent variables in a logistic regression, the AUC was significantly increased when compared to either CEA or type IV collagen alone (*p* = 0.001 and *p* < 0.001, respectively) (Fig. 3a, Table 3). Values of sensitivity, specificity and accuracy for type IV collagen and CEA are presented in Table 4. The calculated optimal cutoff for type IV collagen and CEA in the ROC was 115 and 2.8 ng/ml, respectively. This cutoff for CEA differs from the clinical cutoff of 5 ng/ml.

**Table 3:** Area under the curve (AUC) for collagen IV, CEA and the combination of collagen IV + CEA

Marker	AUC	Standard error	*p* value
Collagen IV	0.806	0.31	–
CEA	0.926	0.19	–
Collagen IV + CEA	0.955	0.14	0.001 and <0.001*

*p values for comparison of AUC-combined marker versus AUC for CEA and collagen IV, respectively

**Table 4:** Specificity, sensitivity, cutoff and accuracy for the calculated values based on the cohort for type IV collagen (COL IV) and the responding values when using the clinically recommended cutoff for CEA

	1-specificity (false positive rate)	Sensitivity (true positive rate)	Cutoff (ng/ml)	Accuracy
CEA calculated	9.3	80.7	2.8	0.86
CEA clinical	0.0	56.4	5.0	0.77
COL IV calculated	34.7	80.9	115	0.72

The type IV collagen levels in the preoperative samples of patients with CLM (*n* = 94) were more frequently elevated (*n* = 76, 80.9 %) than the CEA level (*n* = 53, 56.4 %) (Fig. 3b), when using a calculated optimal cutoff value of 115 ng/ml for type IV collagen and the clinically recommended cutoff of 5 ng/ml for CEA. Of the patients, 93.6 % (*n* = 88) presented with at least one marker above these cutoff levels of 115 ng/ml for collagen IV and the clinical cutoff of 5 ng/ml (Fig. 3b). For CEA, six (6.4 %) patient samples were missing. These six patients all had collagen IV levels above the cutoff.

### Low levels of type IV collagen and CEA are related to high survival

**Fig. 4:**
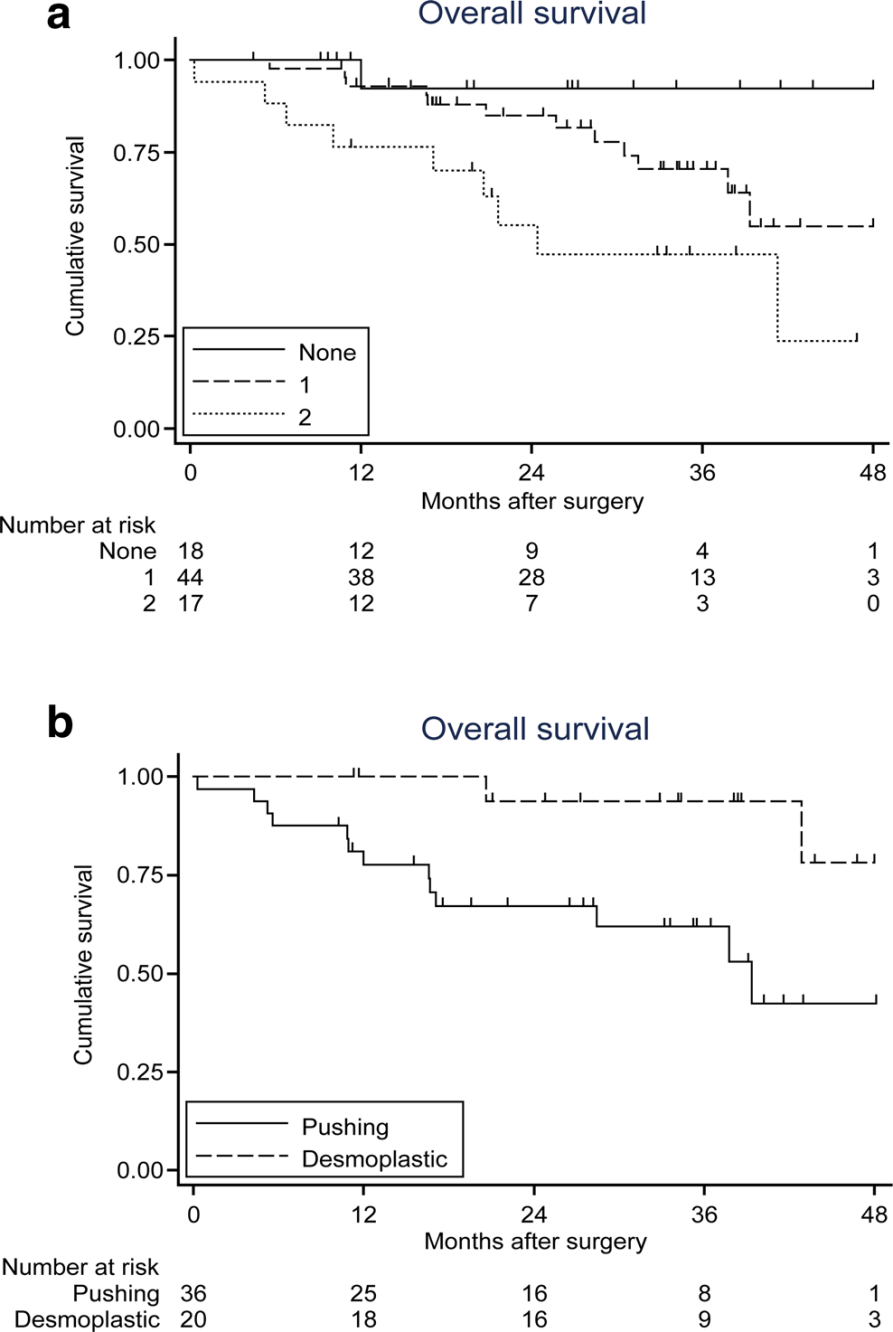
**a** Survival analysis of patients at a median follow-up time of 26.4 months. Grouped as CEA and type IV collagen both below median value (*none*), one tumour marker above median (*1*) and both markers above median (*2*). Patients with both markers above median display the poorest survival. **b** Analysis of survival of patients with the *pushing* versus the *desmoplastic* growth pattern. Patients with a *pushing* type of CLM have significantly poorer survival when compared to the *desmoplastic* type (*p* = 0.011)

To study the prognostic value of a combination of tumour markers, a survival analysis based on both the IV collagen levels and CEA was performed. In this analysis, patients were grouped based on both markers being below the median value, one marker above median or both markers above the median (Fig. 4a). The correlation between the levels of circulating type IV collagen and CEA in relation to mortality revealed that patients with low levels of both markers (*n* = 18) had the best survival (92 %), patients with one marker above median (*n* = 44) displayed a survival of 70 % and patients with both markers elevated (*n* = 17) had the poorest survival of 47 % three years after surgery (Fig. 4a).

### Circulating type IV collagen levels are not related to metastatic pattern of CLM

Two independent observers classified the metastatic growth pattern of the CLM, with an inter-rater agreement of 0.86. The different metastatic growth patterns are illustrated in Supplementary Fig. 1. For 27 patients, no tissue sample was available for analysis due to substantial tumour regression caused by chemotherapy and no viable cancer cells left (*n* = 16), the use of radiofrequency ablation (RFA) (*n* = 4), aborted surgery due to disseminated disease (*n* = 3) or missing samples (*n* = 4). The CLM growth pattern was thus classified in 67 patients (Fig. 1b).

The dominant pattern in each CLM was used for classification, and for the majority of cases (*n* = 58, 87 %), only one pattern was present. In some cases, the CLM were classified as mixed due to features of pushing, desmoplastic and replacement growth pattern (*n* = 9, 13 %). Several of the 67 patients included in the histological analysis had received some form of chemotherapy prior to liver surgery (*n* = 39, 58 %, Table 2). However, the metastatic pattern was still easy to define for most of these patients.

There was no relationship between the major types of CLM (desmoplastic and pushing) and the circulating collagen IV levels. The replacement type of CLM presented with normal type IV collagen levels, but this group was too small to allow for statistical analysis.

To eliminate the possible effects of preoperative oncological treatment on CLM growth pattern, patients that had not received any kind of chemotherapy at least 3 months prior to liver surgery (*n* = 28) were analysed separately (subcohort II). Patient characteristics of subcohort II are shown in Table 1. There was no significant difference in type IV collagen levels between the pushing and the desmoplastic group in subcohort II. The number of patients within the replacement and the mixed group were too small to allow for statistical comparison. Thus, there were no significant differences in the type IV collagen levels between desmoplastic and pushing type of CLM, regardless of whether they had received chemotherapy prior to surgery or not (*p* = 0.632 and *p* = 0.144, respectively).

### The pushing type of CLM is related to poor survival

A Kaplan-Meier analysis of the patients with pushing (*n* = 36) and desmoplastic (*n* = 20) type of CLM revealed that patients with the pushing type had a poorer survival compared to the desmoplastic type of CLM (Fig. 4b). Sixteen patients (44 %) in the pushing group had died compared to two patients (10 %) in the desmoplastic group (*p* = 0.011). Due to few cases of the other metastatic patterns (replacement *n* = 2; mixed *n* = 9), they could not be statistically analysed.

## Discussion

Circulating type IV collagen has been shown to be a potential tumour marker for non-resectable CLM, and the levels of this marker reflect the tumour burden in the liver [6]. Interestingly, patients with primary CRC do not have increased type IV collagen levels, which indicate that the increased type IV collagen levels are related to the liver metastatic process itself. In this study, it is shown that the preoperative circulating type IV collagen levels in patients with resectable CLM are significantly elevated when compared to healthy controls. In addition, patients with recurrent CLM again have increased levels of type IV collagen, whereas patients without recurrence do not.

It is not known why patients with CLM have increased levels of circulating type IV collagen, but earlier studies have shown that CLM are rich in type IV collagen within the metastasis [7, 6]. The elevated levels might be caused by an increased production and/or increased degradation. The increased production seen in CLM may be caused by an increased production by the cancer cells themselves and/or by the myofibroblasts in the liver producing the tumour stroma. In primary CRC that developed CLM, a strong expression of type IV collagen in the vicinity of the cancer cells was seen; this was not observed in primary CRC that never metastasized [7]. A study by Burnier et al. showed that cancer cells with type IV collagen production are highly liver-metastatic and that this trait is lost when their production of this collagen is blocked [14]. This indicates that an upregulation of type IV collagen production in tumour cells might be of importance for the liver-metastatic properties of CRC cells.

In this study, circulating type IV collagen levels were compared to the conventional tumour marker CEA. Type IV collagen was not better than CEA in the ROC analysis when studying either marker alone. However, in this analysis the clinically recommended cutoff level 5 ng/ml is not taken into consideration when calculating the curve. Instead, the cutoff level for CEA by using a ROC curve analysis on the present cohort was estimated to be at 2.8 ng/ml. Thus, there is a discrepancy between the clinically recommended cutoff value for CEA and the cutoff value that results from the ROC curve analysis. This is further underlined by the present data showing that when the clinically recommended cutoff level for CEA (5 ng/ml) was used, circulating type IV collagen was clearly more sensitive in detecting CLM than CEA. Thus, type IV collagen levels were elevated in 81 % of patients with CLM, compared to CEA being elevated in 56 % of patients.

When using the calculated CEA value based on this cohort, sensitivity, specificity and accuracy were better than for type IV collagen. But when using the clinical cutoff at 5 ng/ml, the specificity of CEA was still better than for type IV collagen, but the sensitivity was decreased to 56.4 compared to 80.9 for type IV collagen. Accuracy for both markers was similar. Positive predictive value and negative predictive value are not applicable on this constructed cohort, since these values are dependent on the prevalence of the disease studied.

The combination of both markers resulted in a significantly increased AUC, which indicates that the combination of CEA and type IV collagen improves the sensitivity in detecting CLM when compared to CEA alone (*p* = 0.001). Additionally, patients with both CEA and type IV collagen elevated above the median had a significantly poorer survival (47 %), compared to patients with only one (70 %) or no marker elevated (92 %) 3 years after surgery, implying a prognostic significance using the combination of tumour markers in CLM patients.

Our study has some limitations. First, the controls were younger than the CLM group studied. A gender- and age-matched control group would have been more optimal. A small but significant correlation between age and circulating type IV collagen was observed within the control group. Earlier studies have shown that type IV collagen levels are not related to age [15]; however, the findings in the present study indicate that such a difference might exist but might also be caused by a too small control group with few older individuals. In the CLM group, there was no correlation between age and collagen IV. CEA levels have been reported to relate to age [16], but here, no such correlation was observed for the control group or the CLM group. Another limitation of this study is that follow-up samples were not available from the entire cohort. However, the postoperative samples that were available had quite similar proportions of patients with a recurrent CLM and those without a recurrence. Nevertheless, the present data should be verified in a larger cohort with long-term samples.

In the present study, the correlation of any one of the CLM growth patterns to high circulating levels of type IV collagen was analysed, as there are significant differences in type IV collagen expression among these. However, no such correlation was found. Therefore, preoperative circulating type IV collagen levels are not related to the CLM growth patterns. It was also verified in this study that the pushing type of CLM is associated to poor prognosis as previously described [7–9] with a mortality of 44 %, compared to a mortality of only 10 % in the desmoplastic group.

CLM patients are often diagnosed with their primary CRC prior diagnosis of CLM, and about 50 % of CRC patients will develop CLM. As metastatic disease ultimately is what kills the patients, it is essential to detect resectable metastatic disease early, thus enabling curative metastatic surgery that provides the only hope of lasting cure. There is a great need for better tumour markers allowing for early detection of CLM, as CEA is only elevated in about half of CRC patients. Still only about 22 % of patients with CLM are eligible for resection, and increasing this percentage would provide patients with a chance to actually survive their disease. Type IV collagen in this study has a higher sensitivity for detecting CLM than CEA, and the combination of both markers as shown in this study is significantly better in detecting CLM than using CEA alone (*p* = 0.001). These markers represent different tumour compartments, and measuring substances related to both the cancer cell (CEA) and the stroma (type IV collagen) could provide a better chance to detect CLM than using one marker only.

## Electronic supplementary material

Below is the link to the electronic supplementary material.

Supplementary Table 1 (DOCX 63 kb)

Supplementary Table 2 (DOCX 62 kb)

Supplementary Table 3 (DOCX 17 kb)

Supplementary Figure 1 Reticular (RET) and haematoxylin (H & E) staining can be used to classify the CLM growth patterns. The *desmoplastic* growth pattern is characterized by a dense desmoplastic reaction (DR) separating the tumour cells (T) from the normal liver, with a rich inflammatory infiltrate present in the DR (A, B). The *pushing* growth pattern is characterized by tumour cells (T) pushing the normal liver away with little or no inflammation present (C, D). In the *replacement* growth pattern, the tumour cells (T) grow into the liver parenchyma with a conserved architecture of the normal liver (E, F), and in the mixed growth pattern, multiple growth patterns are present in the same CLM. (DOCX 42 kb)

Supplementary Fig 1 (GIF 602 kb)

High Resolution Image (TIFF 7368 kb)
