# Supplementary material for: Improved tumour marker sensitivity in detecting colorectal liver metastases by combined type IV collagen and CEA measurement
Source: Tumour Biol. 2015 Jul 11;36(12):9839–47. doi: 10.1007/s13277-015-3729-z (PMC4689748; doi:10.1007/s13277-015-3729-z)
Supplement: Supplementary file 1 — (DOCX 63 kb) [file 13277_2015_3729_MOESM1_ESM.docx]

| Point addressed | Description |
| --- | --- |
| **INTRODUCTION**  1 | Fulfilled, marked examined, study objectives, hypothesis. |
| **MATERIALS AND METHODS**  2 Cohort characteristics  3 Treatments  Specimen characteristics  4  Assay methods  5  Study design  6  7  8  9  Statistical analysis  10  11 | Described, inclusion and exclusion criteria specified.  Described.  Described. Sample specification, preservation and storage.  Described, but no validation available for type IV collagen. Assays were blinded to the study endpoint.  Retrospective study, no specific matching between study objects and controls. Other points addressed  Described.  Described.  Not addressed.  Described.  Described. |
| **RESULTS**  Data  12  13  Analysis and presentation  14  15  16  17  18 | Flow of patients described through flowchart.  Demographic of subjects described  Prognostic variables reported related to marker  Logistic regression, ROC, Kaplan Meier, log rank. Further described in manuscript.  Not preformed.  See manuscript.  Not applicable. |
| **DISCUSSION**  19  20 | Preformed.  Addressed. |

**Supplementary table 1.**

The REMARK checklist addressed in this study.
