# Supplementary material for: Improved tumour marker sensitivity in detecting colorectal liver metastases by combined type IV collagen and CEA measurement
Source: Tumour Biol. 2015 Jul 11;36(12):9839–47. doi: 10.1007/s13277-015-3729-z (PMC4689748; doi:10.1007/s13277-015-3729-z)
Supplement: Supplementary file 2 — (DOCX 62 kb) [file 13277_2015_3729_MOESM2_ESM.docx]

**Supplementary table 2A**.

Collagen IV and CEA values in the control group related to age and gender.

|  | **Average** | **Median** | **SD** | **Min-Max** | p-value |
| --- | --- | --- | --- | --- | --- |
| **Collagen IV**  *30-39 (n=6)*  *40-49 (n=16)*  *50-59 (n=32)*  *60-69 (n=62)*  *70-79 (n=2)* | 101.8  72.3  94.6  117.7  110.1 | 93.8  73.0  90.1  117.0  110.1 | 15.5  16.2  32.8  29.7  34.0 | 86.2-130.8  45.1-110.7  42.4-162.0  60.5-239.2  76.8-144.8 | *0.001 |
| **CEA**  *30-39 (n=6)*  *40-49 (n=16)*  *50-59 (n=32)*  *60-69 (n=62)*  *70-79 (n=2)* | 1.03  1.51  1.60  1.46  1.53 | 1.00  1.42  1.34  1.16  1.53 | 0.33  0.68  0.83  0.93  0.72 | 0.62-1.58  0.49-3.24  0.59-3.53  0.22-4.16  0.81-2.25 | 0.460 |

|  | **Average** | **Median** | **SD** | **Min-Max** | **p-value** |
| --- | --- | --- | --- | --- | --- |
| **Collagen IV**  *Male (n=60)*  *Female (n=58)* | 105.5  103.2 | 105.8  96.4 | 33.1  32.7 | 42.4-239.2  43.5-163.0 | 0.979 |
| **CEA**  *Male (n=60)*  *Female (n=58)* | 1.5  1.46 | 1.28  1.3 | 0.85  0.87 | 0.36-4.16  0.22-4.1 | 0.759 |

**Supplementary table 2B**

Collagen IV and CEA values in the CLM group related to age and gender.

|  | **Average** | **Median** | **SD** | **Min-Max** | p-value |
| --- | --- | --- | --- | --- | --- |
| **Collagen IV**  *30-49 (n=6)*  *50-59 (n=13)*  *60-69 (n=47)*  *70-79 (n=26)*  *80-89 (n=2)* | 140.1  189.0  157.3  180.3  244.9 | 142.4  176.6  154.9  160.1  - | 43.5  81.4  61.0  81.8  95.8 | 71.3-192.3  68.3-340.8  65.1-395.5  100.3-411.6  177.2-312.6 | 0.32 |
| **CEA**  *30-49 (n=5*)*  *50-59 (n=13)*  *60-69 (n=45*)*  *70-79 (n=23*)*  *80-89 (n=2)* | 2.84  28.6  70.0  36.8  8.05 | 2.9  7.1  6.6  12.9  - | 1.61  39.3  308.3  63.3  9.0 | 0.8-4.9  1-118  1.1-2073  1.2-300  1.7-14.4 | 0.10 |

** 6 CEA levels were missing*

|  | **Average** | **Median** | **SD** | **Min-Max** | **p-value** |
| --- | --- | --- | --- | --- | --- |
| **Collagen IV**  *Male (n=63)*  *Female (n=31)* | 169.6  167.3 | 160.1  155 | 72.4  69.0 | 65.1-411.6  65.1-340.8 | 0.89 |
| **CEA**  *Male (n=59)*  *Female (n=29)* | 20.4  107.1 | 7.1  10.7 | 31.1  383.8 | 1.1-133  0.8-2073 | 0.35 |
