# Supplementary material for: Improved tumour marker sensitivity in detecting colorectal liver metastases by combined type IV collagen and CEA measurement
Source: Tumour Biol. 2015 Jul 11;36(12):9839–47. doi: 10.1007/s13277-015-3729-z (PMC4689748; doi:10.1007/s13277-015-3729-z)
Supplement: Supplementary file 3 — (DOCX 17 kb) [file 13277_2015_3729_MOESM3_ESM.docx]

**Supplementary table 3**

Levels of circulating type IV collagen correlated to disease recurrence and site of recurrence in subcohort I.

| Patient | Type IV collagen  (ng/ml) | Recurrent disease  (No/Yes) | Site of recurrence |
| --- | --- | --- | --- |
| 1 | 85.9 | No | - |
| 2 | 101.7 | No | - |
| 3 | 66.0 | No | - |
| 4 | 82.0 | No | - |
| 5 | 94.9 | No | - |
| 6 | 66.0 | No | - |
| 7 | 88.0 | No | - |
| 8 | 69.5 | No | - |
| 9 | 99.4 | No | - |
| 10 | 70.9 | No | - |
| 11 | 177.8 | Yes | Liver, lung |
| 12 | 62.44 | Yes | Lung |
| 13 | 142.2 | Yes | Rectal, lymph nodes |
| 14 | 246.6 | Yes | Liver |
| 15 | 171.6 | Yes | Liver |
| 16 | 698.1 | Yes | Liver, lung |
| 17 | 158.7 | Yes | Liver, lung |
| 18 | 198.9 | Yes | Liver, lung |
| 19 | 173.4 | Yes | Liver, lung |
| 20 | 206.2 | Yes | Liver, lung |
| 21 | 199.65 | Yes | Ovarial mass |
| 22 | 246.6 | Yes | Liver |
| 23 | 193.4 | Yes | Liver, bone |
| 24 | 104.74 | Yes | Liver |
| 25 | 130.7 | Yes | Liver |
| 26 | 116.2 | Yes | Liver |
| 27 | 174.0 | Yes | Liver |
