# Supplementary material for: Improved tumour marker sensitivity in detecting colorectal liver metastases by combined type IV collagen and CEA measurement
Source: Tumour Biol. 2015 Jul 11;36(12):9839–47. doi: 10.1007/s13277-015-3729-z (PMC4689748; doi:10.1007/s13277-015-3729-z)
Supplement: Supplementary file 4 — Reticular (RET) and haematoxylin (H & E) staining can be used to classify the CLM growth patterns. The desmoplastic growth pattern is characterized by a dense desmoplastic reaction (DR) separating the tumour cells (T) from the normal liver, with a rich inflammatory infiltrate present in the DR (A, B). The pushing growth pattern is characterized by tumour cells (T) pushing the normal liver away with little or no inflammation present (C, D). In the replacement growth pattern, the tumour cells (T) grow into the liver parenchyma with a conserved architecture of the normal liver (E, F), and in the mixed growth pattern, multiple growth patterns are present in the same CLM. (DOCX 42 kb) [file 13277_2015_3729_MOESM4_ESM.docx]

**Supplementary figure 1**

Reticular (RET) and Haematoxylin (H&E) staining can be used to classify the CLM growth patterns. The *desmoplastic* growth pattern is characterized by a dense desmoplastic reaction (DR) separating the tumour cells (T) from normal liver, with a rich inflammatory infiltrate present in the DR (A, B). The *pushing* growth pattern is characterized by tumour cells (T) pushing the normal liver away with little or no inflammation present (C, D). In the *replacement* growth pattern the tumour cells (T) grow into the liver parenchyma with a conserved architecture of the normal liver (E, F) and in the mixed growth pattern multiple growth patterns are present in the same CLM.
