# Supplementary figures and images for: Improved tumour marker sensitivity in detecting colorectal liver metastases by combined type IV collagen and CEA measurement
Source: Tumour Biol. 2015 Jul 11;36(12):9839–47. doi: 10.1007/s13277-015-3729-z (PMC4689748; doi:10.1007/s13277-015-3729-z)

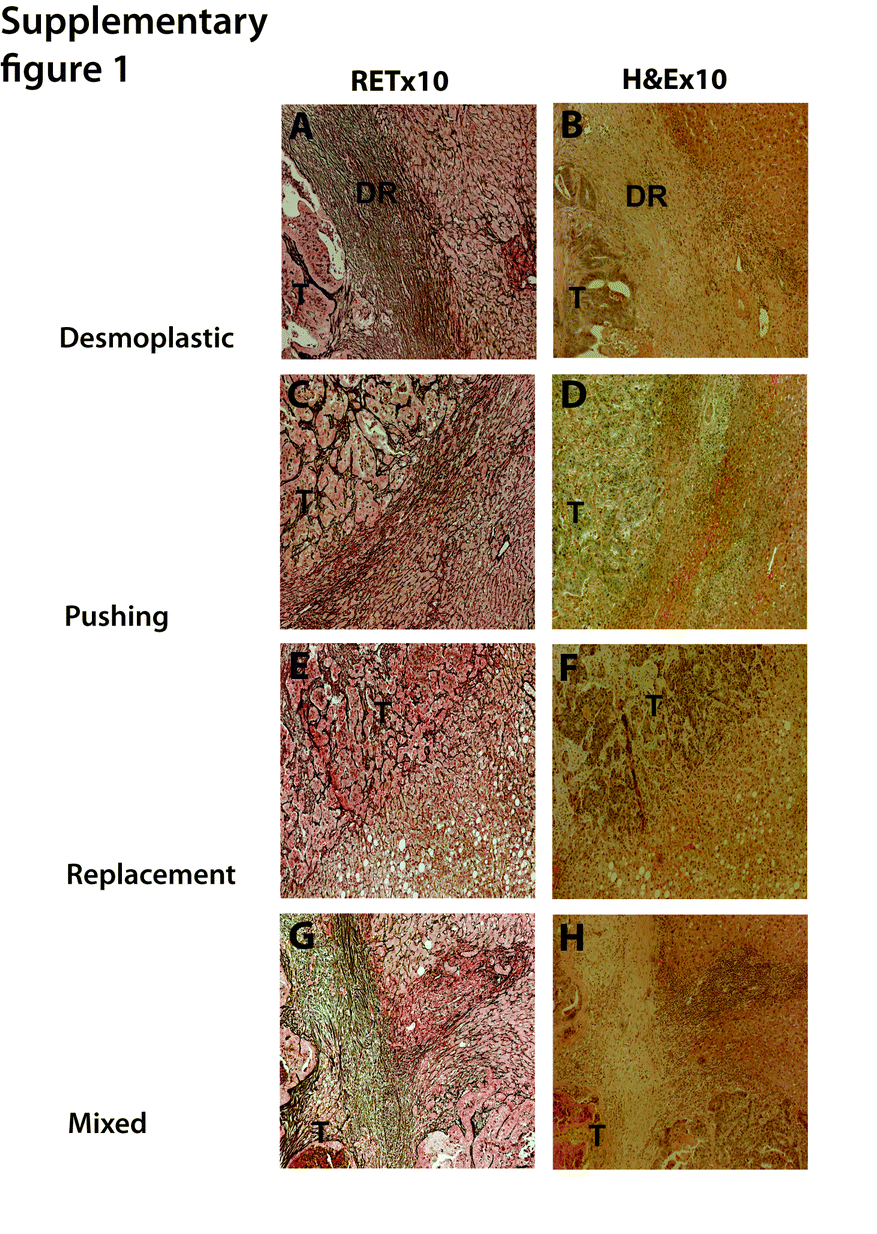

Supplement: Supplementary file 5 — (GIF 602 kb) [file 13277_2015_3729_Fig5_ESM.gif]

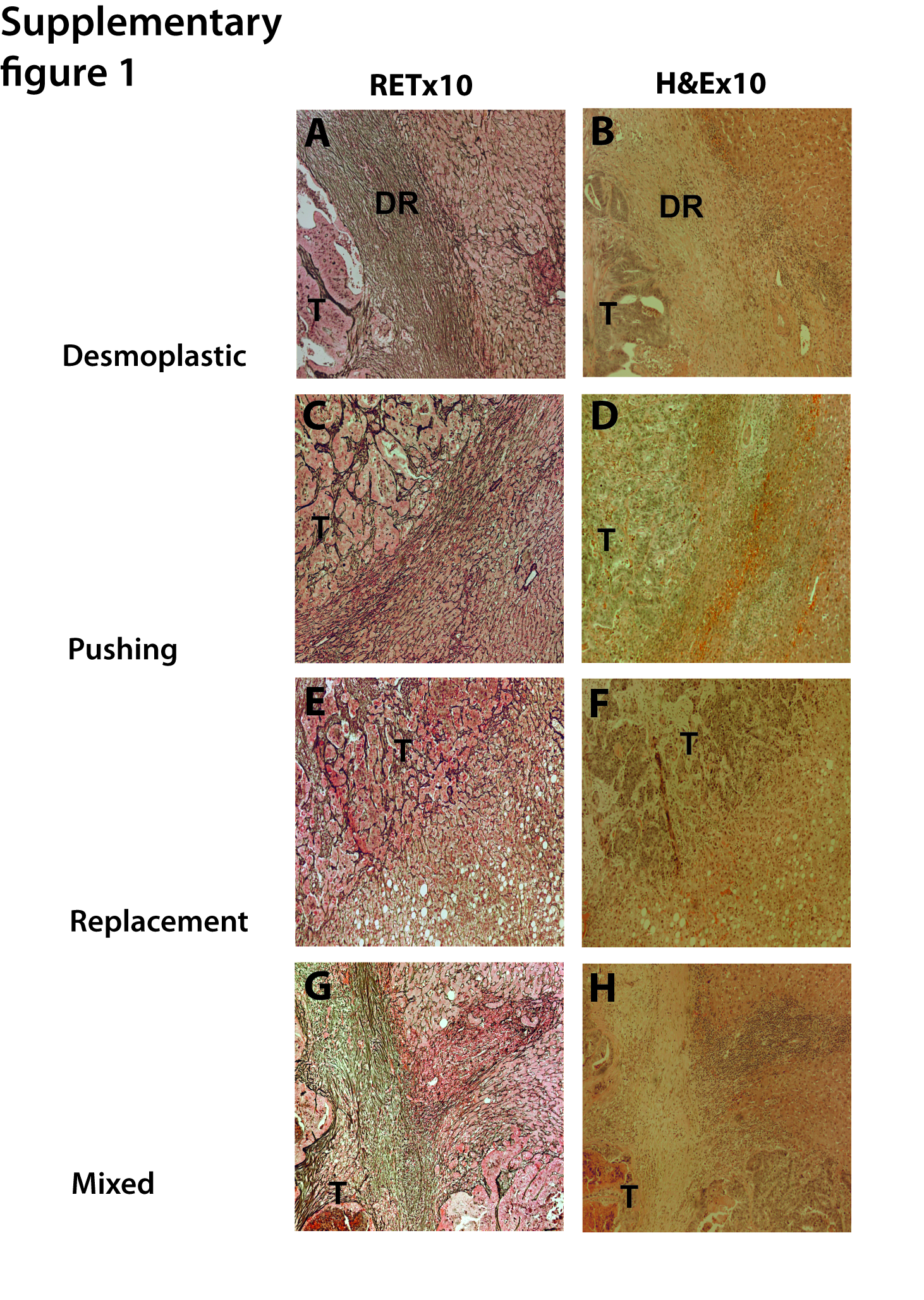

Supplement: Supplementary file 6 — High Resolution Image (TIFF 7368 kb) [file 13277_2015_3729_MOESM5_ESM.tif]
